# National divergence in cardio-kidney-metabolic syndrome burden and implications for health policy: a global burden of disease analysis with projections to 2050

**DOI:** 10.3389/fpubh.2026.1858041

**Published:** 2026-07-15

**Authors:** Lizhi Li, Haoyu Chen, Ping Liu, Zhixiang Jia

**Affiliations:** Kidney Transplantation and Dialysis Center, The Second People's Hospital of Shanxi Province, Taiyuan, China

**Keywords:** cardio-kidney-metabolic syndrome, diabetic kidney disease, global burden of disease, high fasting plasma glucose, ischemic heart disease, non-alcoholic fatty liver disease

## Abstract

**Background:**

The co-occurring epidemics of diabetes and obesity have increased the prevalence of Cardio-Kidney-Metabolic (CKM) syndrome. Comparative studies on long-term trends of its three core components [ischemic heart disease (IHD), diabetic kidney disease (DKD), non-alcoholic fatty liver disease (NAFLD)] across major countries are still limited.

**Methods:**

We analyzed age-standardized disability-adjusted life year (DALY) rates of IHD, DKD, and NAFLD attributable to high fasting plasma glucose (HFPG) from 1990 to 2021 among seven representative middle-and high-income countries using Global Burden of Disease 2021 data. We assessed temporal trends, conducted hierarchical clustering, and projected burdens to 2050.

**Results:**

We found substantial cross-country heterogeneity. From 1990 to 2021, IHD burden decreased significantly in the United States and Japan, while trends in China and India showed high uncertainty (coefficients of variation >100%) and should be interpreted with caution. DKD burden increased in Saudi Arabia and the United States but decreased in China. NAFLD burden increased in Saudi Arabia, the United States, India, and South Africa, while declining in China and Japan. Cluster analysis identified three patterns: “High IHD Burden” (India), “High Metabolic Burden” (Saudi Arabia), and “Low-Moderate Burden” (other countries). HFPG was associated with the largest share of DKD burden (Population Attributable Fraction [PAF] >80%) and a substantially smaller share of NAFLD burden (PAF <11%).Projections to 2050 show a sharp rise in DKD in the United States and India, together with increasing NAFLD burden, indicating a shift toward metabolic organ damage.

**Conclusions:**

The burden of CKM syndrome is substantial and dynamic, with marked cross-country differences. These findings support the need for integrated, multi-organ risk management strategies for metabolic disorders.

## 1 Introduction

The heart, kidneys, and liver are closely linked through shared metabolic pathways. The recently defined Cardio-Kidney-Metabolic (CKM) syndrome highlights interactions among metabolic risk factors, chronic kidney disease, and cardiovascular disease (1). Diabetes affects more than 536 million adults worldwide, and this number is expected to reach 783 million by 2045 (2). The roles of diabetes in ischemic heart disease (IHD) and diabetic kidney disease (DKD) have been well established. Meanwhile, the growing epidemic of non-alcoholic fatty liver disease (NAFLD)—now renamed metabolic dysfunction-associated steatotic liver disease (MASLD)—which affects about 65% of patients with type 2 diabetes, has become a third key component of this syndrome (3). These three conditions form an interconnected network driven by insulin resistance and chronic inflammation (4), supporting a shift from a vasculocentric view of diabetic complications to a more comprehensive multi-organ perspective (5, 6). In this study, we focused on IHD as the main cardiovascular manifestation of CKM syndrome, together with its renal (DKD) and hepatic (NAFLD) components.

Many epidemiological studies have reported trends for individual CKM-related diseases, most often within single countries. For example, cardiovascular disease mortality, especially from IHD, has declined in high-income countries owing to advances in coronary interventions, although progress has slowed amid rising obesity and diabetes rates (2, 7). Meanwhile, the global prevalence of NAFLD rose from 25.26% in 1990–2006 to 38.2% in 2016–2019, making it the most common chronic liver disease worldwide (8). However, few studies have evaluated CKM syndrome as an integrated entity. Systematic comparisons of the joint burden and trends of IHD, DKD, and NAFLD across countries remain scarce. In addition, NAFLD is often overlooked in traditional assessments of diabetic complications. The global variations and future trajectories of CKM burden related to hyperglycemia also remain poorly understood.

To fill these gaps, we conducted a cross-national analysis using the GBD 2021 data across seven representative middle-and high-income countries covering diverse geographic and socioeconomic settings. Our objectives were: (1) to describe temporal trends and age-sex patterns of IHD, DKD, and NAFLD; (2) to quantify the contribution of HFPG to the burden of each CKM component; (3) to identify country-level clusters based on integrated CKM burden profiles; and (4) to project future burden trajectories up to 2050. Our findings aim to provide evidence for developing targeted and integrated strategies for these populations.

## 2 Materials and methods

### 2.1 Study design and data source

This comparative observational study utilized publicly available estimates from the Global Burden of Diseases, Injuries, and Risk Factors Study 2021 (GBD 2021). GBD 2021 provides comprehensive standardized estimates of health loss from 371 diseases and 88 risk factors across 204 countries and territories from 1990 to 2021 (9, 10). This version was selected for its comprehensive and internally consistent time-series data, which supports robust long-term trend analysis and predictive forecasting. All data were de-identified and publicly available. This ecological study is reported in accordance with the STROBE guidelines for cross-sectional studies.

### 2.2 Selection of countries, diseases, and risk factor

To reflect cross-national heterogeneity in CKM syndrome, we selected seven countries based on three predefined criteria. First, geographic representation: we included countries from the Americas (Brazil, USA), East Asia (China, Japan), South Asia (India), the Middle East (Saudi Arabia), and Africa (South Africa). Second, socioeconomic diversity: we selected countries with a high-middle to high Socio-demographic Index (SDI > 0.65 in 2021) to ensure comparable health system capacity and data quality. Third, distinct epidemiological profiles: we included countries with declining cardiovascular mortality (USA, Japan), rapid epidemiological transition (China, India), and high metabolic burden (Saudi Arabia). This design enabled comparative analysis of CKM burden patterns across different stages of the non-communicable disease epidemic. A limitation of this selection strategy is that findings may not be fully generalizable to all middle- and high-income countries, and we acknowledge potential selection bias. Therefore, our results should be interpreted within the context of these seven countries and not extrapolated to low-income settings without further validation. For in-depth demographic analyses, we focused on three representative countries: China, Saudi Arabia, and the United States of America, which exhibited distinct burden profiles.

Countries were selected to represent three key dimensions: (1) geographic diversity, including the Americas, East Asia, South Asia, the Middle East, and Africa; (2) socioeconomic variation, spanning high-middle to high SDI levels; and (3) distinct epidemiologic profiles, including countries with declining cardiovascular mortality, rapid epidemiological transition, and high metabolic burden. This design enabled comparative analysis of CKM burden patterns across stages of the non-communicable disease epidemic.

We focused on three core components of CKM syndrome: IHD, DKD, and NAFLD. High fasting plasma glucose (HFPG) was selected as the primary shared metabolic risk factor.

Our findings are representative of middle-and high-income countries and may not generalize to low-income settings, where health system capacity, infectious disease burdens, and nutritional transitions differ substantially. These contexts warrant dedicated research.

This study used population-level aggregate data from the GBD 2021 study. The units of analysis were the seven selected countries and their populations, rather than individual participants. All available country-level data for these nations and time periods were included; no observations were excluded.

### 2.3 Analytical framework and metrics

Our analysis delivered a multi-dimensional assessment of CKM burden, encompassing temporal trends, demographic patterns, risk attribution, and country-level clustering. The primary outcome measure was the Disability-Adjusted Life Year (DALY). Age-standardized DALY rates (per 100,000 population) were employed for all comparisons to adjust for variations in population age structure. Of note, this study focuses exclusively on age-standardized rates to enable cross-country and temporal comparisons independent of population aging. Absolute DALY counts, which are influenced by population growth and demographic shifts, are not analyzed here. Therefore, a declining rate does not necessarily imply a declining total public health burden if the population is aging or growing. Readers should interpret rate-based trends with this distinction in mind. The contribution of HFPG was quantified using the population attributable fraction (PAF) and HFPG-attributable DALY rates, derived from the GBD 2021 comparative risk assessment framework.

### 2.4 Statistical analysis

We minimized bias by using age-standardized rates to account for confounding attributable to population aging. Standardized data processing and modeling within the GBD framework also improved data comparability across countries and years. Statistical analyses comprised five components that directly corresponded to our research objectives.

#### 2.4.1 Temporal trend and segmented regression analysis

**Figure 1:**
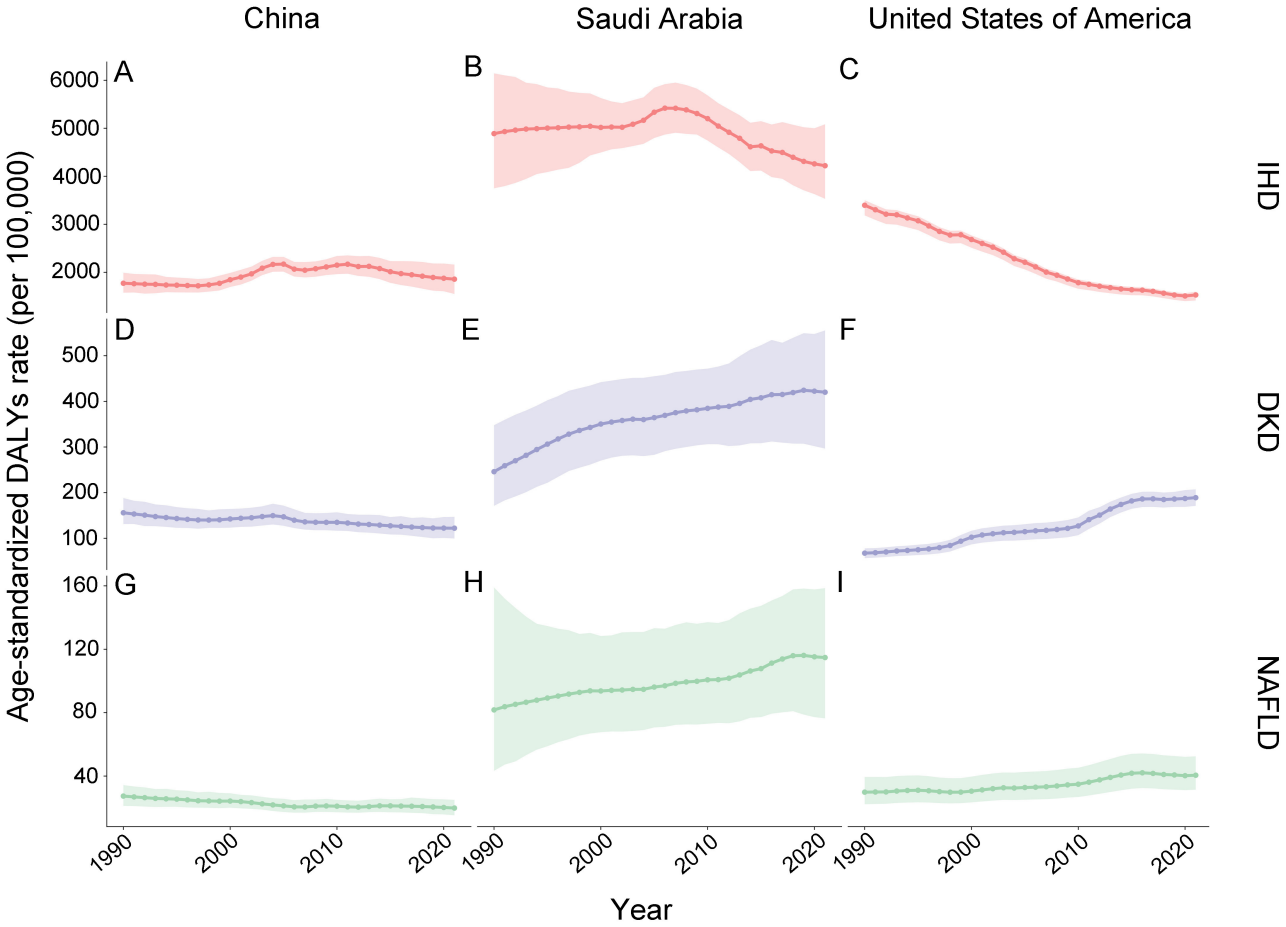
Temporal trends in age-standardized DALY rates for IHD, DKD, and NAFLD among China, Saudi Arabia, and the United States, 1990–2021. Shown are age-standardized DALY rates (per 100,000 population) for the three core CKM components—IHD **(A–C)**, DKD **(D–F)**, and NAFLD **(G–I)**—in three representative countries: China, Saudi Arabia, and the United States of America. Trajectories illustrate key patterns, including declining IHD in the United States of America **(C)**, concurrent increases in DKD in the United States of America and Saudi Arabia **(E, F)**, and rising NAFLD in Saudi Arabia and the United States of America **(H, I)**, in contrast to declining NAFLD in China **(G)**. These findings highlight substantial cross-national heterogeneity in the evolution of CKM syndrome. CKM, cardio-kidney-metabolic; DALY, disability-adjusted life year; IHD, ischemic heart disease; DKD, diabetic kidney disease; NAFLD, non-alcoholic fatty liver disease.

We analyzed temporal trends in age-standardized DALY rates from 1990 to 2021 using a two-step analytical strategy. First, segmented regression (implemented with the R package ‘segmented') was applied to identify significant inflection points, with a pre-specified maximum of two joinpoints. Second, the average annual percentage change (AAPC) was estimated using log-linear regression models to quantify and summarize overall temporal trends. Temporal trajectories for China, Saudi Arabia, and the United States are presented in Figure 1.

#### 2.4.2 Age-sex decomposition

We stratified age-standardized DALY rates by age group and sex for China, Saudi Arabia, and the United States in 2021 to characterize demographic disparities in CKM burden. Population pyramids were employed to visualize these age-sex patterns.

#### 2.4.3 Burden attribution analysis

We extracted population attributable fractions (PAFs) and HFPG-attributable DALY rates for each disease and across all seven countries to quantify the etiological contribution of HFPG. A ternary plot was utilized to illustrate the relative proportional composition of total HFPG-attributable burden.

#### 2.4.4 Burden clustering

Unsupervised hierarchical cluster analysis was performed to identify country groups based on 2021 age-standardized DALY rates for IHD, DKD, and NAFLD. Variables were standardized to Z-scores to ensure equal weighting. Clustering used Euclidean distance and Ward. D2 linkage. The optimal number of clusters (k=3) was determined by visual inspection of dendrograms and clinical relevance.

Sensitivity analyses included comparisons of alternative linkage methods, distance metrics, and cluster numbers (*k* = 2-5). The three-cluster solution remained stable in most sensitivity tests. The clustering analysis included three variables: age-standardized DALY rates for IHD, DKD, and NAFLD in 2021. Ward.D2 linkage was selected because it minimizes within-cluster variance and tends to produce clusters of approximately equal size, which is clinically meaningful for comparing national burden profiles. The choice of *k* = 3 was validated using the silhouette coefficient (average silhouette width = 0.72, indicating strong cluster structure) and the elbow method (see Supplementary Figure S4). Sensitivity analyses using alternative linkage methods (average, complete) and distance metrics (Manhattan) are reported in Supplementary Table S3 and described in the Results (Section 3.7.3).

#### 2.4.5 Future projections

Based on observed 1990–2021 trends, we projected age-standardized DALY rates to 2050 using time-series forecasting models. For primary forecasting, we used the ARIMA (Autoregressive Integrated Moving Average) model with automatic order selection via the auto.arima () function in R (forecast package). All forecasts were generated with 95% prediction intervals over a 29-year horizon to 2050.

To ensure biologically plausible predictions (non-negative rates), we applied log-transformation to all data prior to model fitting, followed by exponentiation of the forecasts.

To evaluate the robustness of our primary forecasts, we conducted a sensitivity analysis comparing three models: ARIMA, exponential smoothing state-space (ETS), and moving average (15-year window). Linear regression was excluded from the ensemble as it can produce negative predictions. Model agreement was quantified using the coefficient of variation (CV), with results presented in Supplementary Table S2.

All analyses were performed using R software (version 4.4.3). Full model specifications and R code are provided in the Supplementary material.

## 3 Results

### 3.1 Temporal trends in the burden of cardio-kidney-metabolic syndromes (1990–2021)

Age-standardized DALY rates for IHD, DKD, and NAFLD in China, Saudi Arabia, and the United States of America from 1990 to 2021 are illustrated in Figure 1. These countries displayed markedly divergent trajectories: the United States of America and Saudi Arabia showed declining IHD burden accompanied by rapidly increasing metabolic organ damage (DKD and NAFLD), whereas China exhibited declines in both DKD and NAFLD burden. These patterns were further validated by segmented regression analysis.

### 3.2 Age- and sex-specific patterns of CKM syndrome burden in 2021

**Figure 2:**
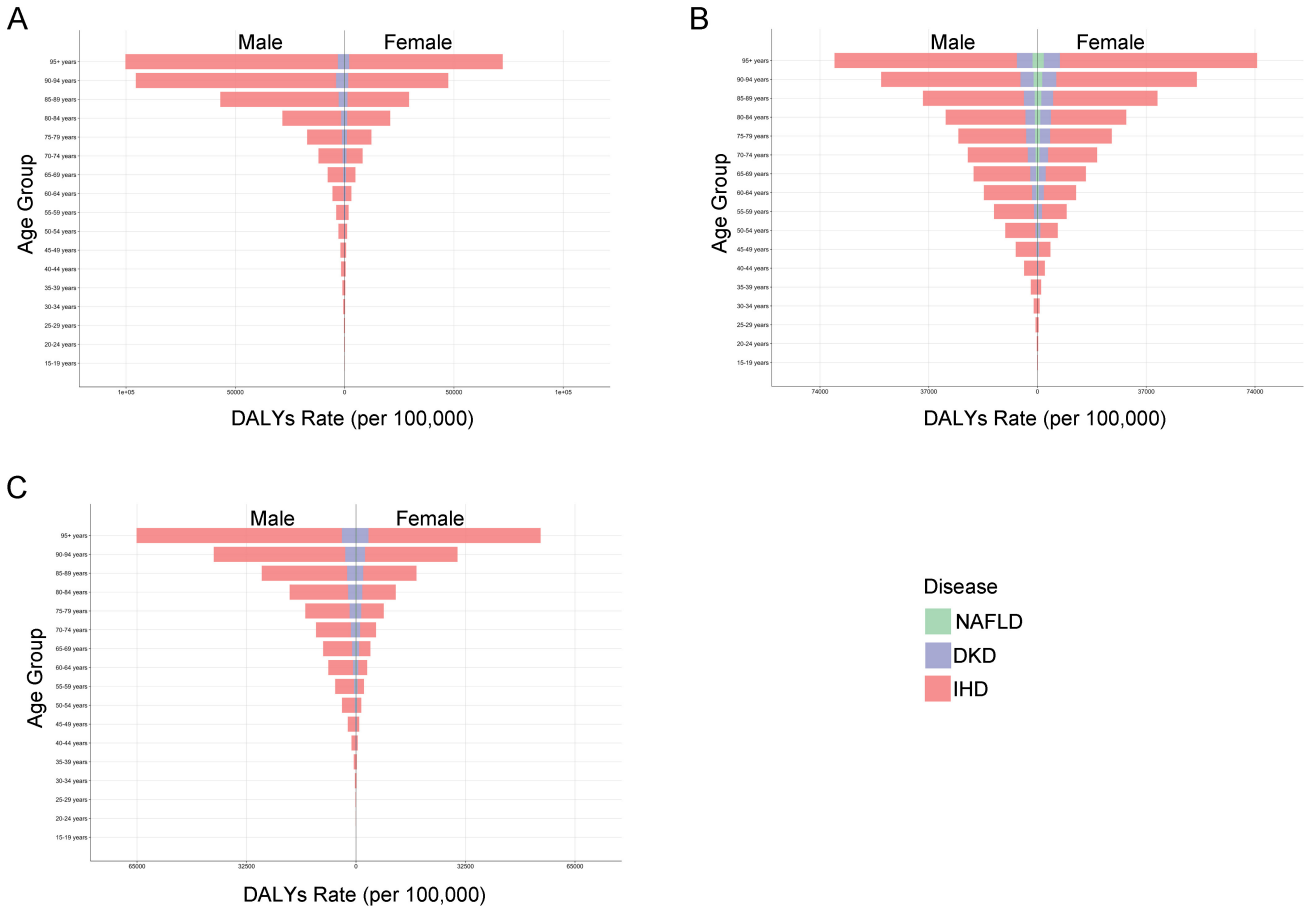
Age- and sex-specific burden of CKM syndromes in 2021. Age- and sex-specific DALY rates (per 100,000 population) for IHD, DKD, and NAFLD are shown for **(A)** China, **(B)** Saudi Arabia, and **(C)** the United States of America. Horizontal bars represent DALY rates by 5-year age group, with males on the left and females on the right of the central axis. Stacked bars show the proportional contribution of each condition to total DALY rates within each age-sex stratum. Notable sex disparities were observed: men carried higher IHD burden across all three countries, whereas women in Saudi Arabia had disproportionately higher DKD and NAFLD burden than men—a pattern distinct from the male predominance seen in China and the United States of America. Abbreviations as in Figure 1.

The age- and sex-specific DALY rates for China, Saudi Arabia, and the United States of America in 2021 are depicted in Figure 2. The burden of all three conditions increased significantly with age and peaked in the oldest age groups. Striking sex disparities were identified: men carried a higher burden of IHD across all three countries, whereas women in Saudi Arabia had higher DKD and NAFLD rates than men—a pattern clearly distinct from the male-predominant burden observed in China and the United States of America.

### 3.3 Cross-country comparison and clustering of CKM syndrome burden

**Figure 3:**
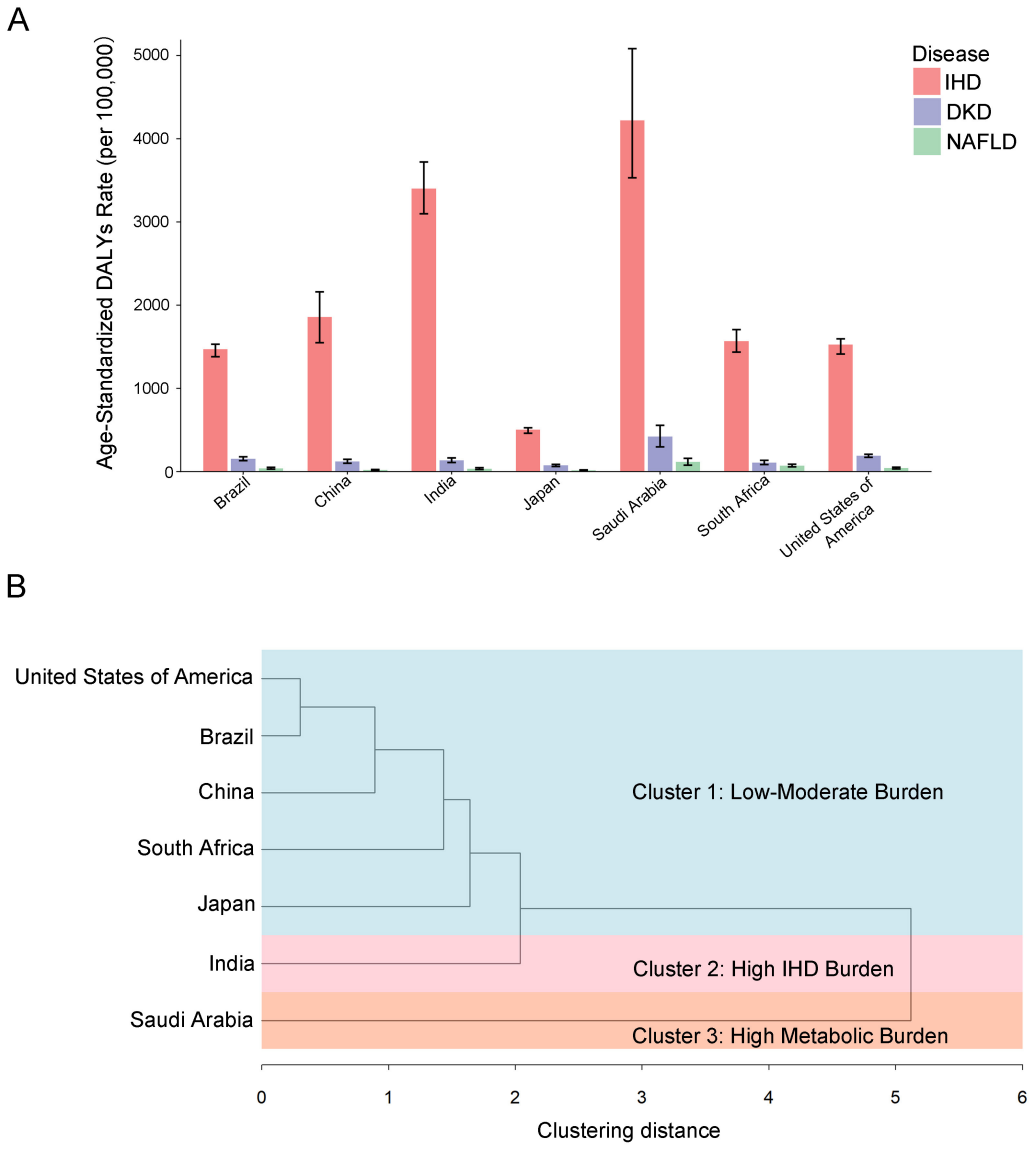
Cross-country comparison and clustering of CKM syndrome burden in 2021. **(A)** Age-standardized DALY rates for IHD, DKD, and NAFLD across seven middle- and high-income countries. Substantial cross-national variation is evident, with India showing the highest IHD burden and Saudi Arabia demonstrating the highest combined DKD and NAFLD burden. **(B)** Hierarchical cluster analysis identified three distinct country clusters: Cluster 1 (Low-Moderate Burden): Brazil, China, Japan, South Africa, United States of America; Cluster 2 (High IHD Burden): India; Cluster 3 (High Metabolic Burden): Saudi Arabia. Interpretation for panel **(B)**: Hierarchical clustering of seven countries based on age-standardized DALY rates for IHD, DKD, and NAFLD. Background colors denote k = 3 clusters: light blue (Cluster 1: Brazil, China, Japan, South Africa, USA), light pink (Cluster 2: India), and light coral (Cluster 3: Saudi Arabia). The bottom axis shows clustering distance. Abbreviations as in Figure 1.

Marked variation in age-standardized DALY rates was observed across the seven study countries in 2021 (Figure 3A). India and Saudi Arabia had the highest total CKM burden. Hierarchical clustering analysis identified three distinct country clusters (Figure 3B): Cluster 1 (Low-Moderate Burden: Brazil, China, Japan, South Africa, the United States of America), Cluster 2 (High IHD Burden: India), and Cluster 3 (High Metabolic Burden: Saudi Arabia).

### 3.4 Risk attribution of cardio-kidney-metabolic syndromes to high fasting plasma glucose

**Figure 4:**
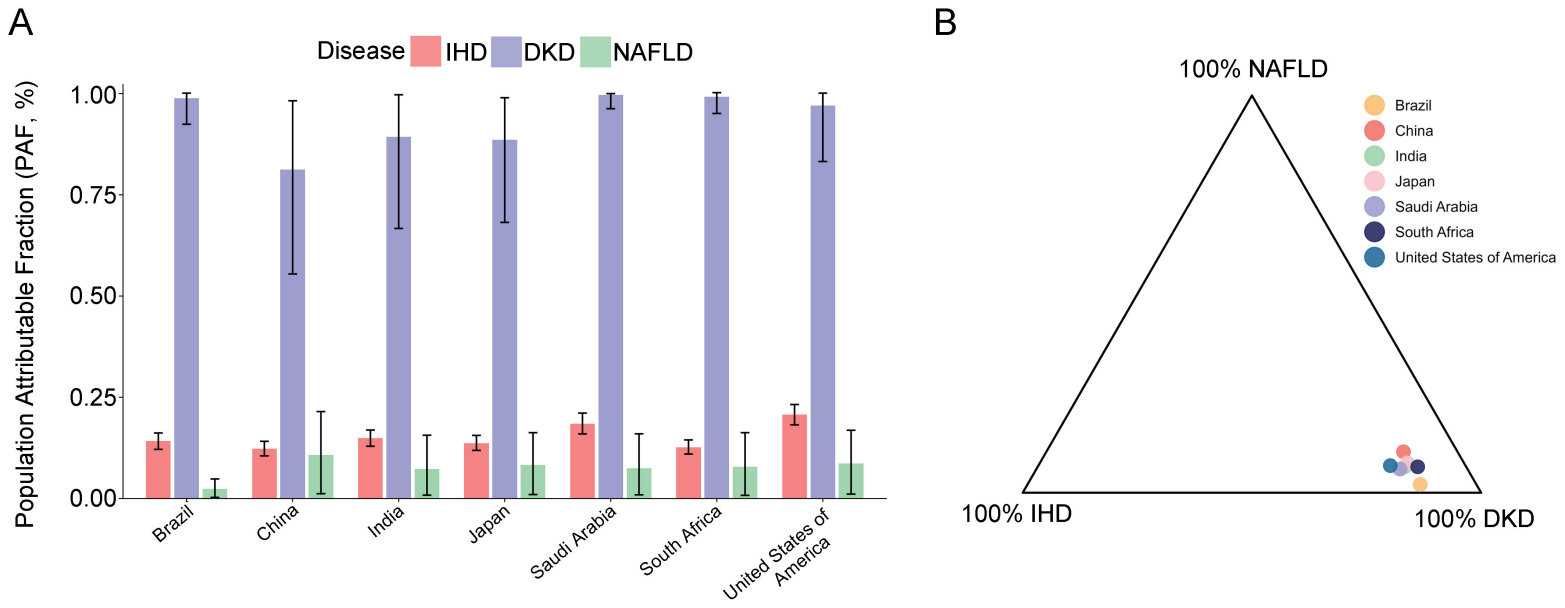
Burden of CKM syndromes attributable to high fasting plasma glucose (HFPG). **(A)** Population attributable fractions (PAFs) of IHD, DKD, and NAFLD attributable to HFPG. HFPG was the dominant contributor to DKD (PAF >89% in all countries), a moderate contributor to IHD (PAF 12%−21%), and a minor contributor to NAFLD (PAF <11%), indicating distinct etiological pathways. **(B)** Ternary plot showing the relative composition of total HFPG-attributable DALYs. Interpretation for panel **(B)**: Each dot stands for one country. Left vertex = 100% IHD, right vertex = 100% DKD, top vertex = 100% NAFLD. Points closer to a vertex represent a higher proportion of HFPG-attributable DALYs for that disease. All countries cluster near the DKD vertex, indicating HFPG drives most DKD burden with minimal contribution to NAFLD. HFPG, high fasting plasma glucose; PAF, population attributable fraction. Other abbreviations as in Figure 1.

The population attributable fractions (PAFs) of IHD, DKD, and NAFLD attributable to HFPG in each country are presented in Figure 4A. Saudi Arabia had the highest PAFs for both IHD and DKD among all study countries. HFPG accounted for the largest share of DKD burden across all nations, with PAF exceeding 89% globally and reaching over 99% in Saudi Arabia. In sharp contrast, the PAFs for HFPG were considerably lower for IHD (range: 12.3%−20.7%) and lowest for NAFLD (range: 2.4%−10.7%). The relative composition of total HFPG-attributable burden for each country, which directly reflects these pronounced disparities, is visualized in the ternary plot (Figure 4B).

### 3.5 Temporal trends and segmented regression analysis

**Figure 5:**
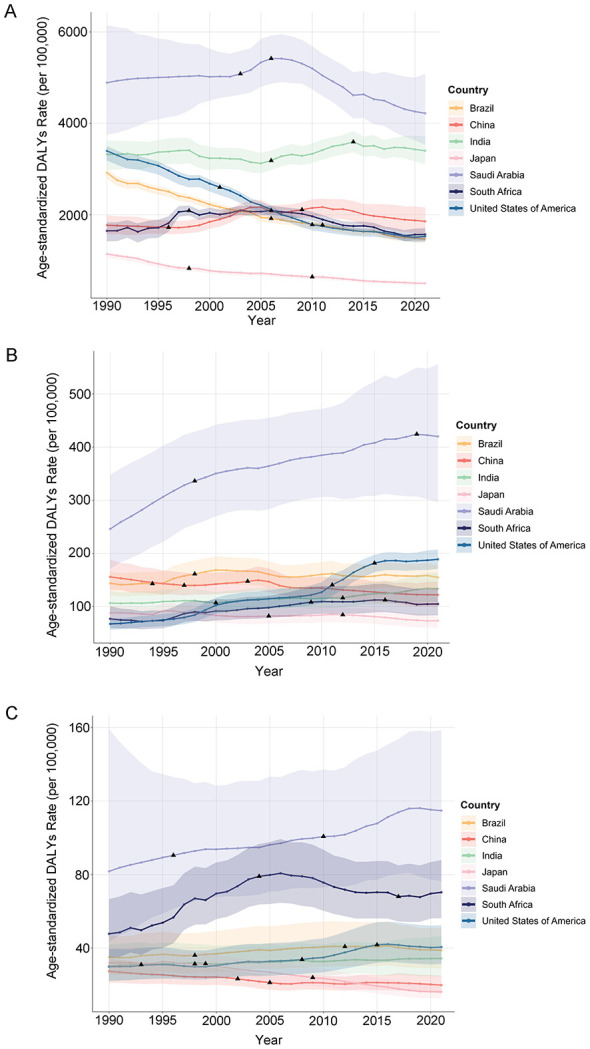
Temporal trends and segmented regression analysis of age-standardized DALY rates for CKM syndromes, 1990–2021. Trends in age-standardized DALY rates for **(A)** IHD, **(B)** DKD, and **(C)** NAFLD across seven countries. IHD burden declined significantly in the United States of America, Japan, and Brazil (AAPC < −2%) but increased in China and India. DKD burden increased in most countries, while NAFLD burden rose notably in Saudi Arabia and the United States of America. Solid lines indicate estimated annual age-standardized DALY rates (per 100,000 population); shaded bands represent 95% uncertainty intervals (95% UI). Triangles (▴) indicate significant change points identified by segmented regression. Summary AAPC values for 1990–2021 are provided in Table 1. AAPC, average annual percentage change. Other abbreviations as in Figure 1.

The long-term trajectories of age-standardized DALY rates from 1990 to 2021 are shown in Figure 5, with significant change points identified by segmented regression marked (▴). The average annual percentage change (AAPC) for each country-disease pair over the full study period is summarized in Table 1.

**Table 1:** AAPC in Age-Standardized DALY Rates for IHD, DKD, and NAFLD from 1990 to 2021.

Country	Disease	AAPC, % (95% UI)	*P* Value	Robustness
Brazil	IHD	−2.16 (-2.24 to−2.09)	**< 0.001**	High
	DKD	0.24 (0.05 to 0.42)	**0.013**	Low
	NAFLD	0.50 (0.39 to 0.62)	**< 0.001**	Low
China	IHD	0.51 (0.24 to 0.78)	**< 0.001**	Low
	DKD	−0.70 (-0.81 to -0.59)	**< 0.001**	High
	NAFLD	−0.97 (-1.12 to−0.83)	**< 0.001**	Medium
India	IHD	0.18 (0.06 to 0.30)	**0.005**	Low
	DKD	0.71 (0.62 to 0.80)	**< 0.001**	Medium
	NAFLD	0.38 (0.34 to 0.41)	**< 0.001**	High
Japan	IHD	−2.51 (-2.66 to−2.37)	**< 0.001**	High
	DKD	−0.51 (-0.64 to−0.38)	**< 0.001**	Low
	NAFLD	−2.43 (-2.66 to−2.21)	**< 0.001**	High
Saudi Arabia	IHD	−0.42 (-0.65 to−0.20)	**< 0.001**	Low
	DKD	1.52 (1.32 to 1.72)	**< 0.001**	High
	NAFLD	1.05 (0.97 to 1.12)	**< 0.001**	High
South Africa	IHD	−0.28 (-0.68 to 0.13)	0.169	Low
	DKD	1.44 (1.19 to 1.69)	**< 0.001**	Low
	NAFLD	1.09 (0.60 to 1.57)	**< 0.001**	Low
United States	IHD	−2.90 (-3.05 to−2.74)	**< 0.001**	High
	DKD	3.80 (3.57 to 4.04)	**< 0.001**	High
	NAFLD	1.26 (1.09 to 1.43)	**< 0.001**	Medium

The AAPC represents the average annual compound rate of change over the study period, estimated from segmented regression analysis. Results are presented as AAPC (%) with 95% uncertainty intervals (95% UI). Statistically significant trends (*P* < 0.05) are shown in bold.

^*^Robustness classification based on coefficient of variation (CV) from sensitivity analyses (Supplementary Table S1): High (CV < 30%), Moderate (CV 30–50%), Low (CV 50–100%), Unreliable (CV >100%). Unreliable estimates are not interpretable and should be disregarded.

AAPC, average annual percentage change; UI, uncertainty interval; IHD, ischemic heart disease; DKD, diabetic kidney disease; NAFLD, non-alcoholic fatty liver disease.

Trend analysis demonstrated that IHD burden decreased significantly in the United States of America (AAPC: −2.90%), Japan (−2.51%), and Brazil (−2.16%), Trends in China (+0.51%) and India (+0.18%) showed high uncertainty (CV >100%) and should be interpreted with caution. For DKD, significant increases were observed in Saudi Arabia (+1.52%), the United States of America (+3.80%), India (+0.71%), and South Africa (+1.44%), whereas a significant decrease was found in China (−0.70%). NAFLD burden rose significantly in Saudi Arabia (+1.05%), the United States of America (+1.26%), India (+0.38%), and South Africa (+1.09%), but fell significantly in China (−0.97%) and Japan (−2.43%). Detailed AAPC results, including 95% uncertainty intervals (95% UI) and statistical significance, are provided in Table 1.

### 3.6 Projected burden of CKM syndromes to 2050

**Figure 6:**
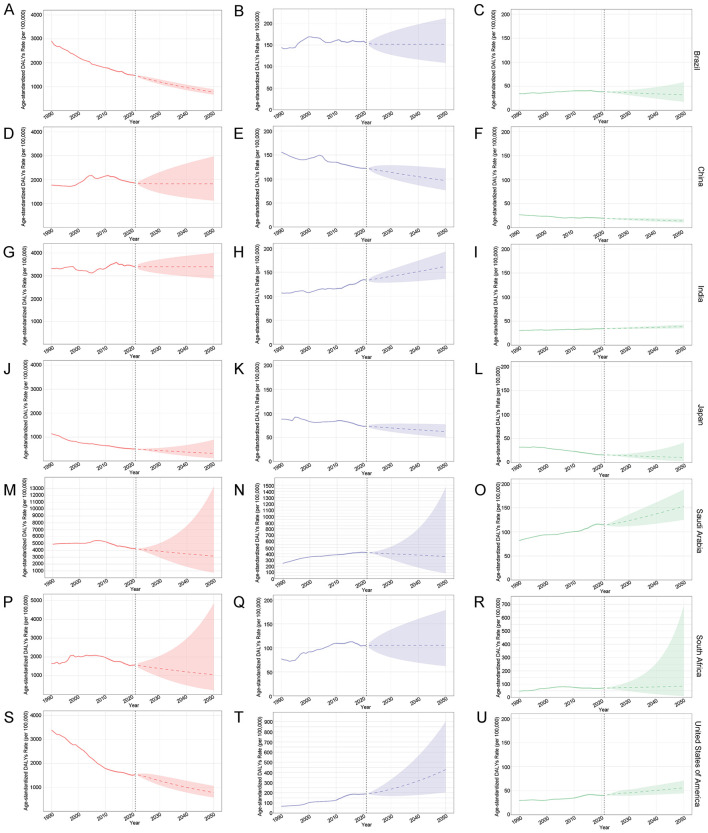
Projected burden of CKM syndromes to 2050. Historical (1990–2021, solid lines) and projected (2022–2050, dashed lines) trajectories of age-standardized DALY rates for CKM syndrome components across seven middle- and high-income countries. Shaded areas represent 95% prediction intervals. Projections indicate a key epidemiologic shift: DKD burden will rise sharply in the United States of America and India, NAFLD burden will continue increasing in several countries, while IHD burden declines. These trends underscore the growing challenge of metabolic organ damage and support the need for a paradigm shift in clinical and public health strategies. Abbreviations as in Figure 1. The figure consists of 21 panels **(A-U)** arranged in a 7-row by 3-column grid. Left column **(A, D, G, J, M, P, S)**: IHD; middle column **(B, E, H, K, N, Q, T)**: DKD; right column **(C, F, I, L, O, R, U)**: NAFLD.

The forecasted age-standardized DALY rates from 2022 to 2050 are displayed in Figure 6. Projections to 2050 underscore a critical divergence in CKM syndrome trajectories. NAFLD burden is projected to continue increasing in Saudi Arabia, India, the United States of America, and South Africa. IHD burden is expected to continue declining in the United States of America, Japan, Brazil, and Saudi Arabia. Most notably, DKD burden is projected to rise sharply in the United States of America (126.6% increase from 2021 to 2050) and India (21.2% increase), while continuing to decrease in China. These divergent trends illustrate a clear shift from macrovascular toward microvascular and metabolic organ damage. Of note, projections for IHD in the United States showed substantial disagreement across sensitivity models (CV = 44.9%), indicating lower reliability. Therefore, these projections should be interpreted with caution.

### 3.7 Sensitivity analyses

#### 3.7.1 Sensitivity of temporal trends and AAPC estimates

Comprehensive sensitivity analyses were performed for segmented regression and AAPC estimation, including variations in joinpoint number (0–3), time windows, bootstrap uncertainty evaluation, and outlier robustness tests. Reliability was assessed using the coefficient of variation (CV). As detailed in Supplementary Table S1, high robustness (CV < 20%) was observed for key trends, including declines in DKD burden in China (AAPC: −0.77%) and Japan (−0.66%), as well as declines in IHD burden in Brazil (AAPC: −2.08%), Japan (−2.44%), and the United States of America (−2.65%). Conversely, substantially higher uncertainty was noted for DKD trends in Brazil (CV: 128%), India (CV: 34.6%), and South Africa (CV: 71.7%), and for IHD trends in China (CV: 872%) and India (CV: 133%). These findings should therefore be interpreted with greater caution.

#### 3.7.2 Sensitivity of forecasts to 2050

The robustness of projections was evaluated by comparing three forecasting models (ARIMA, ETS, moving average). Linear regression was excluded due to its potential to produce invalid negative values. Detailed model comparison results for key country–disease pairs are provided in Supplementary Table S2. Overall, forecasts showed moderate to high robustness for NAFLD (mean CV: 10.5%) and DKD (mean CV: 14.6%), and lower robustness for IHD (mean CV: 25.0%; USA IHD: CV = 44.9%). Consequently, the main conclusions—especially the sharp projected rise in DKD burden in the United States and India—remained consistent across sensitivity analyses.

#### 3.7.3 Sensitivity of clustering analysis

The three-cluster solution (High IHD Burden, High Metabolic Burden, Low-Moderate Burden) was generally stable across sensitivity analyses. Supplementary Table S3 shows that 6 of 7 countries (85.7%) had identical cluster assignments across alternative linkage methods and distance metrics. However, South Africa showed unstable membership: it fell into Cluster 1 (Low-Moderate Burden) under Ward and average linkage but shifted to Cluster 2 under complete linkage. This suggests that South Africa may have an intermediate burden profile that lies between the Low-Moderate and High IHD clusters. Therefore, the assignment of South Africa should be interpreted with caution.

## 4 Discussion

In this comprehensive, cross-national analysis of CKM syndrome, we identified a fundamental and ongoing shift in the profile of diabetes-related complications. Our principal finding is marked heterogeneity in the burden of IHD, DKD, and NAFLD across the seven study countries, which translated into three distinct national clusters with unique demographic characteristics. Furthermore, we demonstrated that although HFPG was the dominant contributor to DKD, its role was moderate for IHD and relatively minor for NAFLD, indicating distinct underlying etiological pathways. Critically, our projections extend these findings to 2050, suggesting a diverging epidemic: the declining burden of IHD is being replaced by the rapid rise of DKD and NAFLD as the primary clinical and public health challenge in several countries, thus requiring an urgent update to current diabetes care strategies.

The evolving burden of the CKM triad identified in this study occurs against a background of well-documented increases in individual metabolic diseases. Recent global reports have quantified the rising burden of type 2 diabetes and MASLD/NAFLD (11), and highlighted their alarming trends among young adults (12). Our study integrates these findings into a unified framework for understanding CKM syndrome. We extend beyond describing separate epidemics to demonstrate their interconnected nature and divergent trajectories—in particular, the key shift from dominant macrovascular (IHD) complications toward increasing microvascular and metabolic organ (DKD, NAFLD) damage. By further clarifying the distinct etiological roles of HFPG across the three conditions and identifying country-specific burden patterns, we offer a strengthened multi-dimensional framework to support the development of targeted and integrated public health and clinical strategies.

The heterogeneity in CKM burden patterns offers a valuable geographical perspective for understanding the global diabetes epidemic. The identification of an “High Metabolic Burden” cluster (represented by Saudi Arabia) reflects a severe cardiometabolic risk profile, likely driven by high prevalence of obesity, metabolic syndrome, and physical inactivity (13, 14). Notably, our demographic analysis shows that women in Saudi Arabia carry a disproportionately high burden of DKD and NAFLD compared with men—a new finding that underscores a unique gender disparity within this cluster and requires further exploration of its underlying causes. Our projections indicate that Saudi Arabia may be at a transition point, with historically high IHD burden starting to decline, while NAFLD burden continues to increase. In contrast, India's classification as a “High IHD Burden” cluster may stem from its rapid epidemiological transition, characterized by early-life factors (e.g., fetal programming, childhood malnutrition), genetic susceptibility to insulin resistance and atherogenic dyslipidemia, and consequent early-onset IHD (15). Worryingly, our model predicts that India will face increasing burdens across all three CKM components, indicating an uncontrolled and comprehensive cardiometabolic crisis.

The marked declines in IHD burden in the United States of America, Japan, and Brazil are consistent with decades of effective public health interventions and medical progress in cardiovascular care (7, 16). However, the simultaneous and projected sharp increase in DKD burden in the United States of America (a projected 126.6% rise by 2050) highlights a major and growing challenge. These findings suggest that although strategies targeting macrovascular disease have been successful, underlying metabolic dysfunction—driven by sustained high prevalence of obesity and diabetes (7, 17)—is now increasingly causing accelerated microvascular end-organ damage. This pattern may be described as the “metabolic legacy effect” of the obesity epidemic, in which the same metabolic dysfunction that causes macrovascular disease also substantially increases the risk of microvascular complications, as reported in recent cohorts (18).

Our quantification of PAFs (Figure 4A) provides critical insights into the distinct etiological roles of HFPG across the CKM triad. The markedly high PAF for DKD is consistent with HFPG being a major contributor to diabetic nephropathy, mechanistically driven by hyperglycemia-induced activation of metabolic pathways leading to oxidative stress and inflammation (19–21). The persistent rise in DKD burden in the United States of America and India thus directly reflects suboptimal glycemic control and the growing prevalence of diabetes. In contrast, the moderate PAF for IHD highlights that hyperglycemia acts within a complex causal network, amplified by hypertension and dyslipidemia. This is supported by clinical trials showing that intensive multifactorial intervention is far more effective than glycemic control alone in reducing cardiovascular events (22, 23). The substantial decline in IHD burden demonstrates the success of strategies targeting these additional risk factors, even amid rising diabetes prevalence.

The low PAF for NAFLD is especially noteworthy. It indicates that NAFLD pathogenesis is driven by a wider range of factors, most notably obesity-related insulin resistance and disordered lipid metabolism, consistent with the well-established ‘multiple-hit' model (24). This concept is further supported by the recent consensus reclassifying NAFLD as metabolic dysfunction-associated steatotic liver disease (MASLD), which explicitly places the disease within the broader spectrum of metabolic dysfunction (25). According to this framework, NAFLD pathogenesis is driven predominantly by insulin resistance and obesity-related metabolic dysfunction. Based on the low PAF observed, mild-to-moderate hyperglycemia appears to play a less direct role in NAFLD burden compared with its role in DKD. However, this does not imply independence, as hyperglycemia and insulin resistance are metabolically interconnected and may exert synergistic effects that are not fully captured by univariate PAF estimates.(26, 27). The substantial and increasing NAFLD burden in the United States of America and Saudi Arabia—despite the low HFPG-attributable fraction—provides strong evidence that the main drivers of the NAFLD epidemic are the broader obesogenic environment and systemic metabolic dysfunction, rather than glycemic status alone.

This pathophysiological principle, which defines NAFLD as a systemic metabolic disorder rather than simply a hepatic complication of diabetes, is key to understanding the NAFLD–diabetes relationship, as emphasized in a recent comprehensive review (28). These findings reinforce that NAFLD in patients with diabetes is not merely a complication but a concurrent manifestation of the global dysmetabolic state (24, 26). Our ternary plot (Figure 4B) provides a clear visual summary of these etiological differences. The strong clustering of all countries near the DKD vertex indicates that hyperglycemia accounts for the predominant share of DKD burden. In contrast, the large distance of all points from the NAFLD vertex offers direct visual evidence that NAFLD burden is largely driven by factors other than hyperglycemia, such as obesity and insulin resistance, based on the low PAF observed, consistent with the multiple-hit model (24, 26). This striking visualization highlights that efforts to reduce the global CKM burden must prioritize DKD prevention and care, while simultaneously adopting a comprehensive metabolic approach to tackle the NAFLD epidemic.

The diverging trajectories of CKM syndrome—with declining IHD burden alongside rising DKD and NAFLD burden—highlight an urgent need for a strategic shift from vasculocentric care to integrated, metabolism-centered management of CKM syndrome. Our findings support a move away from glucose-centric care and highlight three key clinical priorities. Of note, the following clinical recommendations are derived from existing clinical trial evidence and guidelines (cited below) and are not directly tested or evaluated by the present GBD data analysis. They are offered here as interpretative commentary based on the observed burden patterns. First, routine incorporation of hepatic risk stratification into standard diabetes care is essential. This can be accomplished using validated non-invasive tools such as the FIB-4 index or ELF test to screen for significant liver fibrosis (29), especially for identifying high-risk patients with type 2 diabetes, who have a substantially higher prevalence of NAFLD and advanced fibrosis (28). Second, sustained and intensive multifactorial risk reduction is critical, with equal emphasis on blood pressure, lipids, and glucose control, as demonstrated in landmark trials such as the Steno-2 study (22). Third, early and proactive use of organ-protective pharmacotherapy is recommended. The emergence of sodium-glucose cotransporter 2 (SGLT2) inhibitors and glucagon-like peptide-1 (GLP-1) receptor agonists—which provide strong cardiorenal protection and also improve hepatic steatosis and histological features (30–34)—offers an unprecedented opportunity to target multiple components of CKM syndrome simultaneously.

Their multisystem benefits underscore a core principle: intervening on shared metabolic pathways that drive CKM syndrome, a concept strongly supported by recent evidence on the pathophysiological interplay between diabetes and NAFLD (28). This approach is further endorsed by the 2022 ADA/EASD consensus guideline, which recommends these agents as first-line therapy for most patients with type 2 diabetes, especially those with or at high risk of cardiovascular disease, chronic kidney disease, or heart failure, thus formalizing a multi-organ protection–centered treatment paradigm (35).

From a public health perspective, our clustering and forecasting analyses offer a strategic framework for resource allocation. Policies should be tailored to national burden profiles: the United States must prioritize aggressive mitigation of the metabolic drivers underlying its projected surge in DKD and NAFLD. Conversely, India requires a dual approach, strengthening primary cardiovascular care to address its high IHD burden while concurrently curbing the rise of metabolic diseases. Notably, the projected declines in key CKM components in China represent a compelling natural experiment, demonstrating that coordinated public health interventions can favorably alter the trajectory of this syndrome. This is exemplified by China's documented success in reducing age-standardized cardiovascular disease mortality from 286.85 to 245.39 per 100,000 between 2005 and 2020, achieved through a multi-pronged strategy including the establishment of a national chronic disease prevention and control system, community-based risk factor management, and advances in medical technology (36). Assessment of the specific policies driving this success could provide transferable lessons for global CKM syndrome management.

### 4.1 Strengths and limitations

#### 4.1.1 Strengths

This study has several key strengths. First, it uses standardized, long-term GBD 2021 data for a cross-national, integrated analysis of CKM syndrome as a unified entity. Second, it employs hierarchical clustering to identify novel country-level burden patterns. Third, it provides validated projections to 2050, offering evidence for future public health planning.

#### 4.1.2 Limitations

Several limitations should be acknowledged. First, data quality varies across countries. Second, PAF estimates from GBD reflect statistical attribution under specific counterfactual assumptions and should not be interpreted as causal. Residual confounding and ecological fallacy may bias these estimates. Therefore, our use of terms such as”was associated with”or”accounted for”indicates statistical association, not causation. Third, the ecological design precludes individual-level conclusions. Fourth, NAFLD burden is likely underestimated due to underdiagnosis. Fifth, projections cannot account for future therapies or policy changes. Finally, findings are generalizable to middle- and high-income countries only.

## 5 Conclusions

This study provides a comprehensive portrait of the burden of Cardio-Kidney-Metabolic (CKM) syndrome across seven middle- and high-income countries, revealing starkly divergent trajectories among its three core components (IHD, DKD, and NAFLD). We demonstrate that the contemporary challenge posed by diabetes is not a single threat but a shifting triple burden:while ischemic heart disease burden continues to decline in the United States, Japan, and Brazil (with uncertain trends in China and India), it is being replaced by a sharp rise in diabetic kidney disease and non-alcoholic fatty liver disease burden. Given the limited contribution of hyperglycemia to NAFLD, these findings demand a paradigm shift from siloed, vasculocentric care to integrated, multi-organ metabolic risk management strategies. Addressing the evolving CKM syndrome epidemic requires urgent, coordinated, and multi-systemic interventions.

## Data Availability

The original contributions presented in the study are included in the article/Supplementary material, further inquiries can be directed to the corresponding author.
