# Supplementary material for: National divergence in cardio-kidney-metabolic syndrome burden and implications for health policy: a global burden of disease analysis with projections to 2050
Source: Front Public Health. 2026 Jul 15;14:1858041. doi: 10.3389/fpubh.2026.1858041 (PMC13416408; doi:10.3389/fpubh.2026.1858041)
Supplement: Supplementary file 1 [file Table_1.docx]

Supplementary Table S1: Sensitivity Analysis of Temporal Trends（AAPC）- Robustness Summary

| **Disease** | **Country** | **Mean AAPC (%)** | **SD AAPC** | **Robust CV (%)** | **AAPC Range** | **Robustness Rating** |
| --- | --- | --- | --- | --- | --- | --- |
| DKD | Brazil | 0.13 | 0.16 | 128.06 | 0.46 | Low |
| DKD | China | -0.77 | 0.13 | 16.40 | 0.40 | High |
| DKD | India | 0.85 | 0.29 | 34.64 | 0.92 | Medium |
| DKD | Japan | -0.66 | 0.42 | 62.93 | 1.41 | Low |
| DKD | Saudi Arabia | 1.32 | 0.26 | 19.43 | 0.62 | High |
| DKD | South Africa | 1.04 | 0.75 | 71.70 | 2.20 | Low |
| DKD | USA | 3.66 | 0.55 | 14.90 | 1.98 | High |
| IHD | Brazil | -2.08 | 0.15 | 6.96 | 0.33 | High |
| IHD | China | 0.09 | 0.78 | 871.84 | 2.19 | Low |
| IHD | India | 0.17 | 0.23 | 133.56 | 0.97 | Low |
| IHD | Japan | -2.44 | 0.11 | 4.34 | 0.29 | High |
| IHD | Saudi Arabia | -0.73 | 0.58 | 79.71 | 1.59 | Low |
| IHD | South Africa | -0.79 | 0.79 | 100.12 | 2.02 | Low |
| IHD | USA | -2.65 | 0.52 | 19.71 | 1.57 | High |
| NAFLD | Brazil | 0.31 | 0.41 | 131.93 | 1.29 | Low |
| NAFLD | China | -0.79 | 0.30 | 37.50 | 0.78 | Medium |
| NAFLD | India | 0.37 | 0.02 | 4.43 | 0.05 | High |
| NAFLD | Japan | -2.72 | 0.44 | 16.30 | 1.09 | High |
| NAFLD | Saudi Arabia | 1.13 | 0.18 | 15.50 | 0.52 | High |
| NAFLD | South Africa | 0.51 | 0.85 | 167.17 | 2.15 | Low |
| NAFLD | USA | 1.26 | 0.30 | 24.00 | 1.18 | Medium |

Robustness Rating: High (CV < 20%), Medium (CV 20-50%), Low (CV > 50%), Uncertain (mean AAPC near zero)
