# Supplementary material for: National divergence in cardio-kidney-metabolic syndrome burden and implications for health policy: a global burden of disease analysis with projections to 2050
Source: Front Public Health. 2026 Jul 15;14:1858041. doi: 10.3389/fpubh.2026.1858041 (PMC13416408; doi:10.3389/fpubh.2026.1858041)
Supplement: Supplementary file 2 [file Table_2.docx]

Supplementary Table S2: Sensitivity Analysis of Forecasts to 2050 - Detailed Results by Model

| **Disease** | **Country** | **ARIMA** | **ETS** | **MA** | **Mean** | **SD** | **CV(%)** |
| --- | --- | --- | --- | --- | --- | --- | --- |
| DKD | China | 96.6 | 98.3 | 100.5 | 98.5 | 2.0 | 2.0 |
| DKD | India | 162.0 | 183.5 | 158.3 | 167.9 | 13.6 | 8.1 |
| DKD | USA | 428.2 | 205.3 | 612.8 | 415.4 | 204.0 | 49.1 |
| IHD | China | 1,822.6 | 1,384.8 | 2,420.4 | 1,875.9 | 519.9 | 27.7 |
| IHD | India | 3,400.0 | 3,400.0 | 3,630.4 | 3,476.8 | 133.0 | 3.8 |
| IHD | USA | 782.3 | 1,382.7 | 592.4 | 919.1 | 412.5 | 44.9 |
| NAFLD | China | 14.4 | 11.6 | 14.6 | 13.5 | 1.7 | 12.6 |
| NAFLD | India | 38.8 | 39.0 | 38.3 | 38.7 | 0.4 | 1.0 |
| NAFLD | USA | 56.2 | 42.1 | 59.9 | 52.7 | 9.4 | 17.8 |

Note: MA = Moving Average; SD = Standard Deviation; CV = Coefficient of Variation. Values represent age-standardized DALY rates per 100,000 population.

This table presents the sensitivity analysis of forecasts to 2050 using three models (ARIMA, ETS, and Moving Average). Linear regression was excluded because it produced invalid negative predictions. Model agreement is quantified using the coefficient of variation (CV).
