# Supplementary material for: National divergence in cardio-kidney-metabolic syndrome burden and implications for health policy: a global burden of disease analysis with projections to 2050
Source: Front Public Health. 2026 Jul 15;14:1858041. doi: 10.3389/fpubh.2026.1858041 (PMC13416408; doi:10.3389/fpubh.2026.1858041)
Supplement: Supplementary file 3 [file Table_3.docx]

**Supplementary Table S3: Sensitivity of Clustering Analysis Across Different Linkage Methods**

This table shows the cluster assignments for each country using three different hierarchical clustering linkage methods. The consistency across methods demonstrates the robustness of the three-cluster solution.

## Table 1: Detailed Cluster Assignments by Method

Cluster assignments for each country across three linkage methods

| **Country** | **Ward's Method** | **Complete Linkage** | **Average Linkage** |
| --- | --- | --- | --- |
| Brazil | 1 | 1 | 1 |
| China | 1 | 1 | 1 |
| India | 2 | 2 | 2 |
| Japan | 1 | 1 | 1 |
| Saudi Arabia | 3 | 3 | 3 |
| South Africa | 1 | 2 | 1 |
| United States of America | 1 | 1 | 1 |

## Table 2: Cluster Consistency Statistics

Agreement statistics across clustering methods

| **Metric** | **Value** |
| --- | --- |
| Total Countries | 7.0 |
| Perfect Agreement | 6.0 |
| Partial Agreement | 1.0 |
| No Agreement | 0.0 |
| Perfect Agreement (%) | 85.7 |
| Overall Agreement (%) | 100.0 |

## Table 3: Cluster Distribution by Method

Number of countries in each cluster by linkage method

| **Method** | **Cluster 1** | **Cluster 2** | **Cluster 3** |
| --- | --- | --- | --- |
| Average Linkage | 5 | 1 | 1 |
| Complete Linkage | 4 | 2 | 1 |
| Ward's Method | 5 | 1 | 1 |

## Interpretation Notes:

• Perfect agreement: Same cluster assignment across all three methods

• Partial agreement: Agreement between two of the three methods

• High agreement rates demonstrate robustness of clustering solution
